# Oral health associates with frailty in Chinese older adults: a chain mediating model of sleep quality and depressive symptoms

**DOI:** 10.3389/fpubh.2025.1607065

**Published:** 2025-10-27

**Authors:** Lili Chen, Junnan Song, Chunrong Zhou, Dan Su

**Affiliations:** School of Nursing, Anhui Medical University, Hefei, Anhui, China

**Keywords:** oral health, frailty, sleep quality, depressive symptoms, chain mediation, older adults

## Abstract

**Objective:**

With the acceleration of the global aging process, frailty has emerged as a significant public health issue impacting the health of the older adults. Additionally, oral health problems associated with aging require urgent attention. This study aims to examine the association between oral health status and frailty in older adults and to analyze the mediating roles of sleep quality and depressive symptoms to provide recommendations for alleviating frailty.

**Methods:**

This is a population-based cross-sectional study conducted in China from November 2024 to February 2025, involving 345 older adults aged 60 years or older. The study completed measures of sociodemographic information, oral health status, sleep quality, depressive symptoms, and frailty. Pearson correlation analysis tested the correlations between the variables, and chain mediation effects were examined using PROCESS macro (Model 6) with 5,000 bootstrap samples. Statistical significance was set at *p* < 0.05.

**Results:**

This study showed a positive correlation between oral health status, sleep quality, depressive symptoms and frailty (*r* = 0.457 ~ 0.583; all *p* < 0.001), the indirect effects of oral health status on frailty mediated by sleep quality, depressive symptoms were 0.073 (95% CI = [0.002 ~ 0.144]), 0.085 (95%CI = [0.032 ~ 0.148]), the effect mediated by both sleep quality and depressive symptoms was 0.090 (95%CI = [0.049 ~ 0.148]). The total indirect effect value of sleep quality and depressive symptoms between oral health status and frailty was 0.248 (95% CI = [0.161 ~ 0.347]), accounting for 52.54% of the total effect.

**Conclusion:**

Our findings suggest that sleep quality and depressive symptoms are associated with the relationship between oral health status and frailty in older adults, indicating potential intervention targets including oral health care, sleep improvement, and mental health management to assist in slowing the process of frailty.

## 1 Introduction

The global population is aging rapidly, particularly in China. By late 2023, individuals aged ≥ 60 years accounted for 21.2% of the population (1). According to United Nations standards, China has officially entered a moderately aging society, posing major socio-economic challenges (2). Frailty is a common geriatric syndrome, resulting from multisystem functional decline and reduced stress resilience. This leads to physiological, psychological, and social changes, heightening risks for depressive symptoms, dependency, disability and premature mortality (3). Consequently, frailty raises familial caregiving burdens and strains healthcare resources, making it a public health priority (4).

The academic community has explored frailty multidimensionally, addressing pathogenesis, risk factors, early screening and interventions (5). Notably, the development of frailty is dynamic and reversible, emphasizing early risk identification (6). Evidence indicates multiple contributing factors, with nutritional imbalances, depressive states, and physical inactivity recognized as key contributors (7). However, the potential role of oral health as a significant indicator of nutritional status and aging in older adults has received insufficient attention (8). Poor oral health is closely linked to systemic inflammatory responses, increasing risks for chronic diseases like diabetes and cardiovascular disease (9), further accelerating multisystem functional decline. Additionally, studies have shown that oral health factors can serve as independent predictors of frailty in community-dwelling older adults (10). With age, structural changes occur in the oral tissues, including tooth loss, decreased chewing ability, salivary gland hypoplasia, and poor oral hygiene (11). Furthermore, cognitive impairments and low treatment compliance among older adults create a vicious cycle, resulting in eating dysfunction, inadequate nutrition, and physical/mental health issues. These factors can lead to malnutrition and other negative health outcomes, ultimately accelerating frailty. Therefore, poor oral health could be a key factor in the early identification of frailty.

Good sleep quality is crucial for physiological homeostasis and mental health. Older adults, as a special population experiencing a decline in physiological function, often face problems like insomnia, sleep fragmentation, and circadian rhythm disorders (12). Unfortunately, these problems are frequently overlooked. A US cross-sectional study revealed that individuals with poor sleep quality and sleep deprivation have higher risks for oral health issues, such as tooth pain and periodontitis (13). Furthermore, oral dysfunction can worsen sleep quality by affecting nutrition and mood. Sleep disturbances also correlate with frailty. A Chinese meta-analysis (14) indicated that declining sleep quality raises frailty likelihood. Similarly, a cross-sectional study (15) in Greece involving individuals over 65 years old also found a significant association between sleep disorders and frailty. Considering these links between sleep quality, oral health status, and frailty, it is essential to consider sleep quality as a mediating variable.

Depression is a significant public health concern, particularly among older adults (16). Its common clinical features include sleep disorders, somatic fatigue, psychomotor retardation, and emotional apathy (17). If depressive symptoms are not addressed promptly and appropriately, they can gradually worsen, posing a serious threat to the physical/mental health of the older adults and significantly reducing their quality of life. Unfortunately, the internalization of emotional expression and masking of somatic symptoms often lead to the neglect of depressive symptoms, delaying their identification and treatment. This oversight exacerbates declines in social functioning, cognitive abilities, and overall functionality, increasing self-neglect risks (16). Considering the complex interaction between sleep quality and depressive symptoms (18), and a bidirectional Mendelian randomization study (19) revealing the strong correlation between frailty and depression. It can be hypothesized that poor oral health correlates with depressive symptoms through its link to impaired sleep quality, thereby relating to frailty.

As noted above, links exist among oral health status, sleep quality, depressive symptoms, and frailty. However, studies exploring the mediating role of sleep quality and depressive symptoms between oral health status and frailty are lacking. Therefore, the current study aims: (i) to explore whether oral health status is associated with frailty, (ii) to explore whether sleep quality and depressive symptoms mediate the relationship between oral health status and frailty, respectively, and (iii) to explore whether sleep quality and depressive symptoms mediate play a chain mediating role in the relationship between oral health status and frailty.

## 2 Materials and methods

### 2.1 Participants

The data used in this study were collected through a cross-sectional study. From November 2024 to February 2025, the researchers conducted a convenience sample survey in Hefei City, Anhui Province, China, in which 345 older adults were selected as respondents. Inclusion criteria were: (i) aged 60 and older; (ii) no verbal communication barriers, able to answer simple questions clearly; and (iii) informed consent and voluntary participation in the study. Exclusion criteria: (i) severe vision or hearing impairment; (ii) presence of severe cognitive dysfunction or psychiatric illness according to the medical record; and (iii) severe heart, brain, kidney and other organ insufficiency or in the acute onset of disease.

### 2.2 Frailty

Frailty was measured by the Tilburg Frailty Indicator (TFI). It was developed by Gobbens (20) in 2010. In this study, we used the Chinese version of the TFI, which was Chineseized by Chinese scholar Xi Xing (21) and was more applicable to Chinese patients with chronic diseases, and the Cronbach’s *α* of the scale was 0.73, with good reliability and validity. In this study, Cronbach’s α for the scale was 0.739.

### 2.3 Oral health status

The oral health status was assessed using the Oral Health Assessment Tool (OHAT), revised by Chalmers et al. (22), on the basis of the Brief Oral Health Checklist and Chineseized by Wang Jieqiong et al. (23), including the following eight items: lips, tongue, gingival tissue, saliva, natural teeth, dentures, oral cleaning, toothache, each item score is 0 (healthy), 1 (mildly impaired), 2 (unhealthy), total score of 0 ~ 16 points, and a score of 3 or more points for poor oral health, with the higher total score indicating poorer oral health. The Cronbach’s *α* of the scale after Chineseized was 0.710, in this research, the Cronbach’s α for the scale was 0.701.

### 2.4 Depressive symptoms

The 10-item Center for Epidemiological Survey-Depression Scale (CES-D-10) (24) was used to assess depressive symptoms. This scale is a simplified version of the 20-item Depression Scale (CES-D) originally developed by Radloff in 1977. The CES-D-10 maintains the structural validity of the original scale while reducing response time and increasing the response rate. The scale consists of 10 items, with scores ranging from 0 to 3 for each item. Questions such as “Hope for the future” and “I am happy” are reverse-scored, and a score of 10 or higher indicates the presence of depressive symptoms. The CES-D-10 has been widely used to screen for depressive symptoms in the Chinese older adult population, and Cronbach’s *α* for the scale was 0.78, with good reliability and validity (25, 26). The Cronbach’s α for this study was 0.748.

### 2.5 Sleep quality

Sleep quality was measured using the Pittsburgh Sleep Quality Index (PSQI), developed by Dr. Buysse (27), a psychiatrist at the University of Pittsburgh, in 1989. The Chinese version of the PSQI was validated by Liu Xianchen et al. in 1996 (28). The PSQI assesses seven dimensions: subjective sleep quality, time to fall asleep, duration of sleep, sleep efficiency, sleep disorders, use of hypnotic drugs, and daytime dysfunction. Each dimension is scored from 0 to 3 points, resulting in a total score ranging from 0 to 21 points. A score greater than 7 points indicates the presence of sleep disturbances, with higher total scores reflecting poorer sleep quality. The scale demonstrates good sensitivity and specificity, with a Cronbach’s *α* coefficient of 0.84, indicating strong reliability. In our study, Cronbach’α coefficient of PSQI was 0.789.

### 2.6 Sociodemographic variables

We also collected age, sex, educational level, marital status, economic level, smoking and drinking status as the covariates. Educational level was divided into primary school or below, middle or high school, and tertiary or above. Marital status contained married and unmarried (including single, widowed, and divorced). Economic level was assessed by the average monthly income per family member and categorized into three groups: <3,000 yuan (CNY), 3,000 ~ 5,000 yuan (CNY) and >5,000 yuan (CNY). Smoking was defined as smoking at least one cigarette per day for a period of six months or more. Alcohol drinking was defined as having consumed at least one standard drink of alcohol within the past year.

### 2.7 Procedure

The investigators distributed the paper questionnaire on-site and guided the older adults participants in filling it out one-on-one. Once completed, the questionnaires were collected and checked immediately. Any missing items were addressed on the spot. For individuals with low education levels or poor vision, respondents provided assistance in completing the questionnaire. In this study, 368 questionnaires were distributed, and 345 valid responses were collected, resulting in a valid response rate of 93.6%.

### 2.8 Statistical analysis

The data were analyzed and processed with SPSS v27.0 software. First, descriptive analyses were used to describe the information about participants via means (SDs) and percentages. Second, group comparisons between the frailty and non-frailty groups were performed using independent samples t-tests for continuous variables and chi-square tests for categorical variables, and Pearson’s correlation analysis was used to explore the correlations between the variables were explored using the Pearson correlation analysis. Finally, to analyze the chain mediation mechanism, the PROCESS 4.1 macro for SPSS, Model 6, was utilized with 5,000 bootstrap samples. Statistical significance was set at *p* < 0.05 (two-tailed). When the 95% CI did not overlap with zero, a significant mediating effect was observed.

## 3 Results

### 3.1 General information

The descriptive analysis of the demographic information is presented in Table 1. Among the 345 older adults included in the study, 192 (55.7%) were men, and 89.6% were married, with age ranging 60 to 95 years, mean age 69.51 years (SD = 7.54), most of the participants (58.9%) had middle or high school education or above.

**Table 1:** Characteristics of all participants.

Variable	Mean (SD)/Frequency (%)				
	Total (*n* = 345)	Frailty Group (*n* = 123)	Non-frailty Group (*n* = 222)	*t/χ^2^*	*P*
Age, years old	69.51 (7.54)	74.24 (8.01)	66.89 (5.80)	8.954	<0.001
Sex				0.380	0.579
Male	192 (55.7%)	66 (19.2%)	126 (36.5%)		
Female	153 (44.3%)	57 (16.5%)	96 (27.8%)		
Educational level				12.530	0.002
Primary or below	142 (41.1%)	66 (19.1%)	76 (22.0%)		
Middle or high school	152 (44.1%)	44 (12.8%)	108 (31.3%)		
Tertiary or above	51 (14.8%)	13 (3.8%)	38 (11.0%)		
Marital status				13.969	<0.001
Married	309 (89.6%)	100 (29.0%)	209 (60.6%)		
Single, widowed or divorced	36 (10.4%)	23 (6.6%)	13 (3.8%)		
Economic level				9.772	0.008
<3,000 yuan/month	142 (41.2%)	63 (18.3%)	79 (22.9%)		
3,000 ~ 5,000 yuan/month	103 (29.8%)	35 (10.1%)	68 (19.7%)		
>5,000 yuan/month	100 (29.0%)	25 (7.2%)	75 (21.8%)		
Smoking, yes	101 (29.3%)	33 (9.6%)	68 (19.7%)	0.552	0.457
Drinking, yes	136 (39.4%)	43 (12.4%)	93 (27.0%)	1.593	0.207
Oral Health, normal	93 (27.0%)	9 (2.6%)	84 (24.4%)	37.444	<0.001
Sleep quality, normal	212 (61.4%)	49 (14.2%)	163 (47.2%)	37.688	<0.001
Depressive symptoms, normal	287 (83.2%)	83 (24.1%)	204 (59.1%)	33.727	<0.001

SD, standard deviation.

### 3.2 Correlation analysis

Pearson correlation analysis (Table 2) shows that oral health status was positively correlated with sleep quality (*r* = 0.539, *p* < 0.001), depressive symptoms (*r* = 0.457, *p* < 0.001), and frailty (*r* = 0.523, *p* < 0.001). Also, sleep quality was positively correlated with depressive symptoms (*r* = 0.583, *p* < 0.001) and frailty (*r* = 0.505, *p* < 0.001). Depressive symptoms was positively correlated with frailty (*r* = 0.573, *p* < 0.001).

**Table 2:** Correlations among study variables.

Variables	Mean	SD	1	2	3	4
1. Oral health status	4.02	2.09	1			
2. Sleep quality	6.81	3.90	0.539^***^	1		
3. Depressive symptoms	6.11	4.01	0.457^***^	0.583^***^	1	
4. Frailty	3.72	2.84	0.523^***^	0.505^***^	0.573^***^	1

^*^P < 0.05; ^**^P < 0.01; ^***^P < 0.001. SD, standard deviation.

### 3.3 Mediation analysis

**Figure 1:**
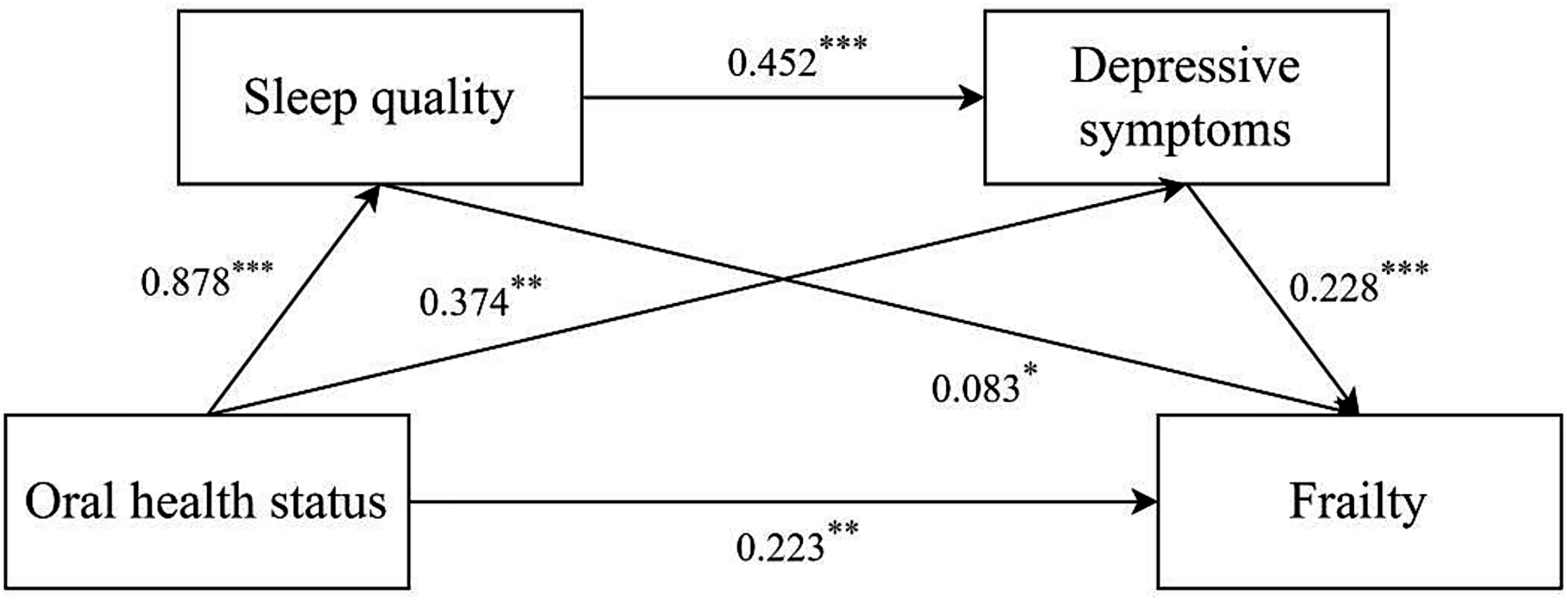
Mediating effect analysis of sleep quality/depressive symptoms between oral health status and frailty. ^*^p < 0.05; ^**^p < 0.01; ^***^p < 0.001.

The chain mediating effect of sleep quality and depressive symptoms between oral health status and frailty is illustrated in Tables 3, 4. Based on the mediation model, the direct effect was 0.223 (95% CI:0.091 ~ 0.335), and the total indirect effect was 0.248 (95% CI:0.161 ~ 0.347). The indirect effects of oral health status on frailty via sleep quality, depressive symptoms, and both were 0.073 (95%CI:0.002 ~ 0.144), 0.085 (95%CI:0.032 ~ 0.148), and 0.090 (95%CI:0.049 ~ 0.148), respectively. The percentage of the three pathways in the total effect was 15.46, 18.01 and 19.07%, respectively (see Table 4). The final path model was shown in Figure 1.

**Table 3:** Mediating roles of sleep quality and depressive symptoms between oral health status and frailty.

Variables	*R^2^*	*F*	*t*	*P*
Outcome: sleep quality	0.347	22.315		
Oral health status			9.061	<0.001
Outcome: depressive symptoms	0.384	23.194		
Oral health status			3.465	0.001
Sleep quality			8.292	<0.001
Outcome: frailty	0.544	39.777		
Oral health status			3.331	0.001
Sleep quality			2.267	0.024
Depressive symptoms			6.836	<0.001

Intermediation analysis under controlling for age, sex, educational level, marital status, economic level, smoking and drinking status.

**Table 4:** Indirect effect of the mediating mode.

Effect types	SE	LLCL	ULCL	Effect	Effect size
Total effect	0.065	0.343	0.600	0.472	100.00%
Direct effects	0.067	0.091	0.355	0.223	47.46%
Total indirect effects	0.047	0.161	0.347	0.248	52.54%
Indirect effects (sleep quality)	0.036	0.002	0.144	0.073	15.46%
Indirect effects (depressive symptoms)	0.029	0.032	0.148	0.085	18.01%
Indirect effects (sleep quality and depressive symptoms)	0.026	0.049	0.148	0.090	19.07%

Intermediation analysis under controlling for age, sex, educational level, marital status, economic level, smoking and drinking status.

## 4 Discussion

In this study, older individuals aged 60 years and older were selected for the cross-sectional analysis. The research aimed to explore the interrelationship and mechanism between oral health, sleep quality, depressive symptoms and frailty in the older adults. The findings revealed a direct correlation between oral health status and frailty in older adults, with the direct effect accounting for 47.46% of the total effect. Additionally, sleep quality and depressive symptoms served as mediators between oral health status and frailty, with the total mediation effect accounting for 52.54% of the total effect; sleep quality and depressive symptoms had the chain mediation effect between oral health status and frailty, accounting for 19.07% of the total effect.

### 4.1 Association between oral health status and frailty

This study found that poorer oral health status is associated with a higher degree of frailty (effect = 0.472, 95% CI = [0.343 ~ 0.600]), consistent with the findings of other research. As early as 2012, scholars Castrejon-Perez et al. (29) observed concurrent higher frailty rates among older adults reporting poor oral health. They also observed that the risk of frailty increased among older adult patients who were hospitalized for oral diseases within the past year. They proposed a hypothesis that oral health may influence frailty through inflammatory, nutritional, and psychosocial pathways. In this study of 345 older individuals, 73% of older adults (*n* = 252/345) exhibited poor oral health per OHAT criteria (score ≥3), indicating a serious oral health problem among older adults. As age increases, the wear and loss of teeth become more pronounced, leading to chronic oral conditions such as dental caries and periodontal disease. Additionally, the older adults often take multiple medications due to comorbidities; adverse reactions from some of these drugs, such as reduced saliva secretion, can impair their oral pH regulation, promote bacterial growth, and give rise to various oral health problems (30). Furthermore, factors such as a limited ability to acquire new knowledge, a lack of oral health-related information, and unhealthy lifestyles can exacerbate oral health issues (31). Tooth loss and oral pain can result in chewing dysfunction and decreased appetite, leading to changes in dietary habits, limited nutrient intake, and low-energy diets. These patterns associate with reduced physical health and increased fatigue, concurrently observed with frailty progression. Furthermore, frailty shows bidirectional correlations with poorer oral health status, suggesting a cyclical relationship.

### 4.2 The mediator role of sleep quality

Our findings suggest that oral health correlates with frailty levels through associations with sleep quality (effect = 0.073, 95% CI = [0.002 ~ 0.144]). As individuals age, their oral immune function weakens, making the gingival and periodontal tissues more susceptible to pathogens. Oral pain, gingival swelling, bleeding, xerostomia, and other symptoms can lead to difficulties in falling asleep, frequent awakenings, and sleep fragmentation in the older adults. Furthermore, tooth loss, especially when multiple teeth are missing, increases the risk of sleep disturbances by 4% for each missing tooth (32). This loss can significantly affect chewing ability, limit food choices, and reduce overall nutritional intake. The resulting discomfort during meals, malnutrition, and poor eating experiences can lead to nighttime hunger and gastrointestinal issues, concurring with diminished sleep quality. Declining sleep patterns demonstrate connections to heightened frailty vulnerability. One study (33) had shown that both excessively long and short sleep durations can lead to hormonal changes, metabolic abnormalities, and inflammation, all of which heighten vulnerability to frailty. Insomnia, in particular, is closely linked to increased body inflammation, insulin resistance, and oxidative stress, which can disrupt protein synthesis and contribute to sarcopenia, exhibiting correlation with frailty (34, 35). Additionally, a cohort study of 3,844 older Japanese subjects found that insomnia not only caused frailty but also exacerbated its symptoms (36). Therefore, oral health status may relate to frailty through interconnected pathways by affecting sleep quality. This suggests that we can mitigate the progression of frailty in older adults by improving both oral health and sleep quality.

### 4.3 The mediator role of depressive symptoms

Our findings suggest that oral health status correlates with frailty levels through associations with depressive symptoms (effect = 0.085, 95% CI = [0.032 ~ 0.148]). Depressive symptoms and oral diseases, both significant public health issues, share a bidirectional relationship. In a meta-analysis, Cademartori et al. (37) found a positive association between depressive symptoms and the risk of oral diseases, including caries, periodontal disease, and tooth loss. These oral health problems often accompany pain and discomfort, adversely affecting an individual’s daily life, including sleep, eating, and speech functions. Moreover, they can lead to changes in facial appearance, impacting self-esteem and self-confidence, and causing embarrassment and feelings of inferiority in social situations, which may reduce social engagement. Additionally, oral treatment tends to be expensive, increasing the economic burden on individuals and negatively impacting mental health, potentially leading to depression. Depressive emotions can trigger inflammatory responses, such as elevated levels of C-reactive protein (38). Chronic inflammation may damage cellular function, accelerate organ aging, and raise the risk of chronic diseases like cardiovascular disease and diabetes (39, 40). Depression can also result in emotional indifference among the older adults, manifested as diminished interest in daily activities, including physical exercise and social interactions, as well as neglect of their health needs, such as delayed medical attention and failure to follow medical advice (41). This neglect can lead to a decline in physical function and a deterioration of overall health. Furthermore, depressed older individuals often experience a loss of appetite, which can result in long-term malnutrition, further weakening their physical function and increasing the likelihood of frailty (42). Consequently, oral health conditions may indirectly contribute to increased frailty by influencing depression levels.

### 4.4 The chain mediating role of sleep quality and depressive symptoms

This study also investigated the chain mediation effect of sleep quality and depressive symptoms (effect = 0.090, 95% CI = [0.049 ~ 0.148]). These results suggest that poor oral health conditions are associated with frailty in the older adults by reducing sleep quality, which is correlated with depressive symptoms. Studies have shown a close relationship between decreased sleep quality and depressive symptoms (43, 44). A meta-analysis (45) found that individuals with insomnia who are not depressed have twice the risk of developing depression compared to those without sleep difficulties. Sleep deprivation can disrupt the neuroendocrine system, leading to decreased levels of neurotransmitters such as serotonin and dopamine, which increases the likelihood of experiencing a depressed mood (46). Additionally, there is a bidirectional relationship between sleep quality and depressive symptoms (18): long-term insomnia can worsen depression, while depression can fragment sleep structure, creating a vicious cycle. In conclusion, the influence of poor oral health may result in decreased sleep quality in the older adults, leading to reduced emotional regulation, stress system disorders, and eventually depressive symptoms. The presence of these depressive symptoms is further correlated with an increased risk of frailty in the older adults.

### 4.5 Limitations

This study has several limitations. First, it utilized a cross-sectional design, which cannot establish a causal relationship between variables. Second, variables such as “sleep quality,” “depression symptoms,” and “degree of frailty” rely on participants’ self-reported data, which may introduce information bias and affect the accuracy of the results. Third, the samples were obtained from only one region of China. Given the lifestyle, economic, and socio-cultural differences across regions, caution should be exercised in interpreting our findings.

## 5 Conclusion

This study aimed to investigate associations between oral health status and frailty in older adults and to examine the mediating effects of sleep quality and depressive symptoms. The findings confirmed that poorer oral health is associated with higher levels of frailty in the older adults. Additionally, oral health shows associations with frailty through interconnections with sleep quality and depressive symptoms. Our results offer guidance for improving the health of older adults by preventing and alleviating the occurrence and progression of frailty. This can be achieved through oral health screenings, regular community clinic activities, and the dissemination of knowledge on proper tooth brushing and denture maintenance. It is essential to enhance older adults’ awareness of oral health care and their self-management abilities. To address sleep disorders, we propose a comprehensive program that includes sleep health education, optimization of the sleep environment, and non-drug interventions. This may involve personalized sleep assessments and promoting regular routines to improve circadian rhythm disturbances in the older adults. On a psychological level, we recommend depression screening, establishing mental health records, and utilizing professional psychological counselors for group counseling, particularly for widowed individuals and empty-nesters, to help them rebuild social connections. By integrating the three pillars of oral health care, sleep management, and psychological support, the government can collaborate with primary medical institutions, family caregivers, and social organizations to effectively delay the progression of frailty and enable older adults to achieve the “aging without frailty” life state.

## Data Availability

The raw data supporting the conclusions of this article will be made available by the authors, without undue reservation.
